# From Criticism to Comfort: The Relational Benefits of Long‐Term Care Insurance

**DOI:** 10.1111/1475-6773.70026

**Published:** 2025-08-13

**Authors:** Xianhua Zai

**Affiliations:** ^1^ Max Planck Institute for Demographic Research Rostock Germany; ^2^ Max Planck – University of Helsinki Center for Social Inequalities in Population Health Rostock Germany; ^3^ Max Planck – University of Helsinki Center for Social Inequalities in Population Health Helsinki Finland

**Keywords:** aging, longitudinal data, long‐term care, network, relationship

## Abstract

**Objectives:**

The objective of this study is to examine whether potentially eligible individuals with Partnership Long‐Term Care Insurance (PLTCI) program experience stronger social networks and improved interpersonal relationships compared to those without coverage.

**Study Setting and Design:**

Our analysis utilizes data from the Health and Retirement Study (HRS), a longitudinal survey of U.S. adults aged 50 and older, incorporating responses from the Leave‐Behind Questionnaire administered biennially from 2004 to 2018. We merge these data with a dataset tracking state‐level implementation of the PLTCI program, enabling us to construct a binary indicator of policy exposure based on respondents' state of residence. Using ordinary least squares (OLS) regression with two‐way fixed effects, we estimate the effect of the PLTCI program on the relational outcomes of aging individuals.

**Data Sources and Analytic Sample:**

The analytic sample includes HRS respondents potentially eligible for the PLTCI program at the time of its implementation, focusing on respondents and their spouse no more than 65 years without physical limitations per Activities of Daily Living (ADL) criteria. Depending on data availability, the sample size ranges from approximately 13,000 to 17,000 participants.

**Principal Findings:**

The PLTCI program improved perceived relationships with children and spouses. Older adults reported less frequent criticism (4.3% decrease with children, *p* = 0.04, 95% CI: 0.3%–8.3%; 3.4% with spouse, *p* = 0.04), feeling let down (3.9% decrease with children, *p* = 0.01; 3.8% with spouse, *p* = 0.009), or being annoyed (3.5% decrease with children, *p* = 0.03). They also felt more comfortable opening up about worries (2.1% increase with children) and relying on close family members during serious problems (3.0% increase with children, *p* = 0.01). These effects were strongest among individuals aged 55 and older compared to younger individuals, non‐Hispanic White respondents compared to non‐Hispanic Black respondents, and those with higher household wealth compared to those with lower household wealth.

**Conclusions:**

Beyond financial security, the PLTCI program enhances older adults' social and emotional well‐being by improving close relationships. These findings highlight the need to consider both economic and relational outcomes when evaluating long‐term care policies.

**Summary**

- What is known on this topic?
  ○ Public long‐term care (LTC) policies, such as the Partnership Long‐Term Care Insurance (PLTCI) program, have been shown to influence older adults' well‐being, particularly by providing financial security and reducing the risk of institutionalization.
  ○ Previous research highlights the importance of LTC policies in supporting both formal and informal caregiving dynamics, but their effects on social relationships and daily interactions remain unclear.
- What this study adds?
  ○ This study provides novel evidence on how the PLTCI insurance program strengthens social networks by improving relationships with close family members, particularly children and spouses.
  ○ It demonstrates that PLTCI coverage reduces interpersonal strain and enhances emotional support, especially among individuals aged 55–65, white respondents, and those with higher household wealth, illustrating the broader social benefits of LTC policies beyond financial security.

## 1 Introduction

The need for a deeper understanding of the impact of public long‐term care (LTC) policies on the well‐being of aging individuals is crucial, especially in terms of their daily activities and social interactions. While much has been discussed regarding the financial aspects of LTC policies [1, 2, 3, 4], the effect on the personal and relational lives remains largely unexplored. This study aims to fill this gap by investigating how the Partnership Long‐Term Care Insurance (PLTCI) program enhances key social networks for aging individuals.

Medicaid is the primary provider of LTC services for eligible older Americans, offering nursing home care and home and community‐based services [5]. While Medicaid eligibility is determined by strict financial and functional criteria, those who fail to qualify face steep costs—approximately $108,405 annually for nursing home care or $30,125 for home health aide services. To mitigate these costs and reduce reliance on Medicaid, the PLTCI program was established, encouraging the purchase of private long‐term care insurance (LTCI) through collaborations between state governments and private insurers. The program features a “dollar‐for‐dollar” asset protection model, allowing policyholders to shield assets equal to their insurance payouts from Medicaid's spend‐down requirements [5, 6, 7, 8]. Initially piloted in four states during the late 1980s and 1990s (California, Connecticut, Indiana, and New York), the PLTCI program expanded significantly following the Deficit Reduction Act of 2005, which encouraged states to implement their own programs.

Between 2006 and 2016, implementation occurred in phases: 3 states adopted the program in 2006, 9 in 2007, 11 in 2008, 8 in 2009, 3 in 2010, 2 in 2011, 1 in 2012, and a final state in 2016 (see Supporting Information: Table A1 for details). This staggered rollout across states presents a valuable opportunity to assess how PLTCI implementation may influence the quality of relationships within individuals' key social networks.

The PLTCI program was designed to increase LTCI coverage by offering Medicaid assets protection to policyholders, making LTCI more accessible and affordable. By integrating private insurance with public safety nets, the program aimed to incentivize responsible LTC planning and reduce the financial risks of LTC expenses [9]. The program mainly benefited wealthier individuals in increasing LTCI coverage [8], while a rise in insurance applications is reported, suggesting an increase in LTCI purchase [7]. In addition, the PLTCI program led to a modest increase in LTCI coverage, particularly among those already inclined to purchase non‐partnership policies [10].

However, there is limited evidence in the literature on how LTCI improves the quality of life for individuals who are aging and may require LTC services as they grow older. Several challenges need to be addressed to answer this question. First, quality of life is difficult to measure, which is why previous literature has focused on life satisfaction [11], physical health [12], informal care [13], financial strain [1], mental health [14], and the health of spouses [15]. However, daily interactions with key networks, such as spouses and children, make up the major daily activities of individuals and are crucial to their quality of life. The lack of empirical evidence on this important aspect needs to be addressed. Second, detailed questions about relationships with key networks are not easily accessible. Most research on relationship‐related topics has used interviews with limited sample sizes and qualitative methods [16, 17, 18]. In this paper, we address this gap by combining a large LTCI program with nationally representative data to form a larger, more representative sample of the aging population. We examine how their relationships with key networks, such as spouses and children, improve due to potential coverage under LTC policies.

### 1.1 LTCI and Relationship Improvement With Key Network

LTCI has the potential to significantly enhance the relationship between aging individuals and their children and spouses by addressing challenges associated with caregiving expectations and reducing the emotional strain on family dynamics. By providing financial and logistical support for care needs, LTCI can mitigate tensions, promote autonomy, and improve communication, ultimately fostering more positive intergenerational and spousal relationships [5, 13, 19].

First, LTCI reduces the reliance on informal caregiving, which often falls on children and spouses. Without insurance, children may feel obligated to provide care [20], leading to stress and potential resentment over the demands on their time, finances, and emotional resources. Similarly, spouses often bear the primary caregiving burden, which can result in significant physical and emotional strain [15, 21, 22], particularly when LTC needs become extensive. These dynamics can strain relationships, as older individuals may sense criticism, reluctance, or frustration from their children or spouses. By having LTCI, individuals can lessen their dependence on family members, allowing caregiving to be a choice rather than an obligation. This shift can lead to more positive interactions, as care provided out of love rather than duty fosters healthier relationships. For instance, research shows that older individuals often expect their children or spouses to provide care, avoiding LTCI purchase [19, 23], which could inadvertently heighten family tensions.

Moreover, LTCI addresses concerns about fairness and caregiving expectations. Within families, the uneven distribution of caregiving responsibilities among children can lead to conflicts and perceived imbalances [24, 25, 26]. Spouses, too, may feel unfairly burdened, especially if they have limited external support. By securing professional care services through LTCI, older individuals can alleviate these pressures. This reduces the likelihood of disputes about caregiving duties among children and lessens the disappointment or criticism spouses might express when care expectations exceed their capacity. Ensuring access to professional care can maintain the dignity of older individuals while preserving the emotional well‐being of their family members.

Finally, LTCI supports the autonomy and self‐reliance of individuals, enhancing their relationships with both children and spouses. When aging adults invest in LTCI, they demonstrate a proactive commitment to managing their own care needs and minimizing the caregiving burden on their loved ones [13, 14]. For children, this can reduce feelings of guilt and foster a sense of mutual respect, as parents take control of their care planning. For spouses, this autonomy can mitigate the physical and emotional toll associated with prolonged caregiving [12, 19, 27], creating space for more meaningful and supportive interactions. Furthermore, the availability of LTCI‐funded care can lead to open and constructive family discussions about LTC preferences and planning, aligning with shared values such as autonomy and avoiding undue burdens [28].

In summary, by reducing dependence on informal caregiving, addressing fairness and caregiving expectations, and promoting autonomy, LTCI serves as a foundation for improving relationships between aging individuals, their children, and their spouses. It allows families to focus on connection and support rather than the logistical and emotional challenges of caregiving, ultimately enhancing their overall quality of life.

### 1.2 The Current Study

Leveraging data from the Health and Retirement Study, this research examines the effects of PLTCI program implementations on the quality of relationships of potentially eligible individuals with their key networks, such as spouses and children.

Drawing from an extensive review of the scientific literature and the theoretical frameworks explored previously, the following hypotheses are proposed for investigation:

Hypothesis 1 *Individuals in states that have implemented PLTCI programs will experience less negative relationships with their key networks compared to those in states without such programs*.

Hypothesis 2 *Individuals in states with PLTCI programs will experience more positive relationships with their spouses and children compared to those in states without PLTCI programs*.

## 2 Methods

### 2.1 Data Source and Study Sample

The Health and Retirement Study (HRS) is a longitudinal survey designed to collect comprehensive data on older Americans, focusing on various aspects of health, economic status, and well‐being. As part of its data collection, HRS includes a unique component known as the Leave‐Behind Questionnaire. This self‐administered survey was piloted in 2004 and has been fully implemented biennially from 2006 to 2018. The questionnaire is distributed to respondents after they complete their core face‐to‐face interviews, covering six key areas: subjective well‐being, lifestyle and stress experiences, quality of social relationships, personality traits, and work‐related and self‐related beliefs. The HRS employs a rotating panel design, which divides participants into two random 50% subsamples. Subsample A receives the Leave‐Behind Questionnaire in 2006, 2010, 2014, and 2018, while Subsample B completes it in 2008, 2012, and 2016. Both spouses or partners in eligible households are invited to participate, providing valuable dyadic data from individuals aged 50 and older. The survey includes not only non‐institutionalized self‐respondents but also proxy respondents and nursing home residents who complete their interviews in person. The data collected from the 2004 pilot study is also included in our analysis, as it provides relevant variables for the study period, allowing us to use data spanning from 2004 to 2018 [29].

The dataset for the PLTCI program provides extensive data on when states implemented the program (Supporting Information: Table A1), making it possible to study how this policy affects individuals' relationships with key members of their social networks, like children, spouses, and friends. This dataset was merged with respondents' state of residence from the HRS, enabling us to define a binary indicator of PLTCI exposure. This indicator reflects whether respondents were exposed to the policy based on its adoption timing relative to HRS survey periods [5].

For our study sample, we identified HRS respondents who potentially met eligibility criteria for the PLTCI program at the time of its implementation. Specifically, we included individuals who were likely to meet the health insurance carriers' prescreening requirements, focusing on those and their spouses who were no more than 65 years of age and without any physical limitations according to the ADL criteria during the HRS study period.

Supporting Information: Figure A1 shows the distribution of ages potentially eligible for the PLTCI program before its implementation. For example, Ohio implemented the program in 2007, and HRS respondents and their spouses who were 65 years old or younger in 2007 were potentially eligible for the program. Additionally, Supporting Information: Figure A2 displays the distribution of actual ages of the study sample included in the analysis from 2004 to 2018.

Based on available, non‐missing outcome data, the sample size ranges from approximately 13,000 to 17,000 participants.

### 2.2 Measures

#### 2.2.1 Dependent Variables

The dependent variables in our analysis are derived from a series of questions designed to evaluate the quality of respondents' social relationships with their spouse or partner, children, immediate family, and friends. These questions assess both positive and negative aspects of social interaction. The positive aspects focus on the extent to which individuals feel understood and supported by those close to them. Respondents are asked how much they believe their spouse, children, or friends truly understand the way they feel about things, how much they can rely on them in times of serious problems, and how much they can open up to them when they need to talk about their worries. Conversely, the negative aspects focus on the extent to which individuals feel criticized, let down, or irritated by their relationships. Respondents are asked how much they experience criticism, how often they feel let down when counting on someone, and how much they are bothered or “get on their nerves” by their relationships. These questions were only asked to participants who had previously reported having a spouse, children, family member, or friends. For these questions, respondents provided their answers using a four‐point Likert scale. To make the interpretation of results easier, the original scale was rescaled as follows: 1 (“Not at all”), 2 (“A little”), 3 (“Some”), and 4 (“A lot”).

#### 2.2.2 Independent Variable and Heterogeneity Dimensions

The main independent variable in this analysis was a binary indicator reflecting whether the PLTCI program was active in the respondent's state at the time of their HRS interview. To control for unobserved factors potentially linked to both LTCI coverage and social network relationships, the model incorporated fixed effects for both the year and the PLTCI‐expansion group.

The decision to use PLTCI‐expansion group fixed effects instead of state fixed effects was made to increase the precision of our estimates, particularly for the heterogeneous results. This is because several states have sample sizes with fewer than 50 observations, which can increase the uncertainty of the heterogeneous estimates and reduce the precision of these estimates. By grouping states with similar implementation years together, we can create larger subsamples with more robust estimates, thereby improving the overall precision of our results; however, we also conduct robustness checks using state fixed effects for the main results.

For a closer look at the nuanced effects on relationships within key social networks, we analyzed variations across four dimensions: age, gender, race/ethnicity, and household wealth.

#### 2.2.3 Analytic Strategy

Descriptive statistics were presented for the dependent variable, comparing individuals who were potentially eligible for the PLTCI program (PLTCI = 1) with those who were not (PLTCI = 0). To analyze how PLTCI implementation influenced the quality of relationships within key networks, OLS two‐way fixed‐effects models were employed in Stata 17. These models incorporated year fixed effects to control for nationwide economic fluctuations and PLTCI‐expansion‐group fixed effects to account for demographic and policy differences. The identification strategy relied on the assumption that the timing and location of PLTCI program implementation were effectively random. Identification drew on variation in PLTCI policy rollout across states and over time, with the assumption that these differences were uncorrelated with unobserved factors specific to each PLTCI‐expansion group. Standard errors were clustered at the state level, with statistical significance indicated as follows: **p < 0.05; ***p < 0.01.

## 3 Results

In states with PLTCI programs, respondents tend to be slightly older, have greater household wealth, and generally report better relationships with both their children and spouses compared to those in states without such programs (see Table 1). For the questions about how often children or spouses criticize them, let them down when they are counting on them, and get on their nerves, lower average scales indicate fewer negative experiences. Respondents in PLTCI states report around a 0.1 unit decrease in these negative experiences with their children compared to those in non‐PLTCI states. Similarly, for relationships with spouses, there is about a 0.1 unit reduction in how often they feel criticized or annoyed, while feeling let down remains relatively similar across groups. On the other hand, higher average scales for questions about being able to open up about worries, rely on their children or spouse when facing a serious problem, and feeling understood indicate stronger emotional support. In PLTCI states, relationships with children show a small but positive increase of about a 0.06–0.09 unit difference in these areas, while the effects on spousal relationships are smaller.

**TABLE 1:** Descriptive statistics of demographics and main variables by PLTCI status in 2004–2018.

	PLTCI = 0			PLTCI = 1		
	*N*	Mean	SD	*N*	Mean	SD
Demographics of individuals						
Age	1,975	55.60	4.71	13,105	61.16	7.88
Female	1,975	0.62	0.48	13,105	0.60	0.49
Non‐Hispanic Black	1,864	0.14	0.35	11,722	0.18	0.38
Household wealth	1,975	419,000	868,000	13,105	569,000	1,510,000
Relationship with children						
Criticize	1,973	1.99	0.91	13,131	1.85	0.90
Let You Down	1,970	1.76	0.81	13,093	1.66	0.78
Get on Nerves	1,970	1.85	0.83	13,259	1.75	0.84
Open up Worries	1,977	3.04	0.93	13,124	3.10	0.91
Rely on Problems	1,977	3.29	0.91	13,146	3.38	0.85
Understand Feelings	1,976	3.10	0.83	13,137	3.16	0.80
Relationship with spouse						
Criticize	1,808	2.14	0.88	11,422	2.04	0.90
Let You Down	1,810	1.98	0.89	11,405	2.01	0.88
Get on Nerves	1,813	1.77	0.82	11,419	1.67	0.83
Open up Worries	1,811	3.44	0.79	11,427	3.46	0.77
Rely on Problems	1,812	3.77	0.60	11,433	3.75	0.59
Understand Feelings	1,810	3.31	0.78	11,416	3.33	0.78

*Note:* The table presents descriptive statistics for the working sample of HRS individuals who were age eligible (no more than 65) and health eligible (no Activities of Daily Living [ADL] limitations) during the period in which the Partnership Long‐Term Care Insurance (PLTCI) program was in place, between 2004 and 2018. Due to the small sample sizes of Hispanic and non‐Hispanic individuals of other races in the non‐PLTCI states, we excluded these groups from the working sample. Only non‐Hispanic White (White) and non‐Hispanic Black (Black) individuals are included in the final working sample. Each dependent variable measures a specific aspect of respondents' relationships: *Criticize* (“How much do they criticize you?”), *Let You Down* (“How much do they let you down when you are counting on them?”), *Get on Nerves* (“How much do they get on your nerves?”), *Open Up Worries* (“How much can you open up to them if you need to talk about your worries?”), *Rely on Problems* (“How much can you rely on them if you have a serious problem?”), and *Understand Feelings* (“How much do they really understand the way you feel about things?”). Responses are on a four‐point Likert scale from 1 (“Not at all”), 2 (“A little”), 3 (“Some”), and 4 (“A lot”).

Table 2 presents the estimated effects of PLTCI implementation on the quality of respondents' relationships with key social network members, based on a specification including PLTCI‐expansion‐group and year fixed effects. Panel A focuses on relationships with children, while Panel B examines relationships with spouses. In Panel A, PLTCI implementation reduced the frequency of children criticizing respondents by 0.09 units (significant at the 5% significant level), representing a 4.3% decrease relative to the mean value of 1.99 in control states without PLTCI. Similarly, the frequency of children letting respondents down decreased by 0.07 units (3.9%) compared to the mean of 1.76 (significant at the 5% significant level), while the annoyance caused by children (“getting on nerves”) declined by 0.07 units (3.5%) against a mean of 1.85 (significant at the 5% significant level). On the positive side, PLTCI improved respondents' ability to open up about worries to their children by 0.07 units (2.1%) relative to the mean of 3.04 (significant at the 10% significant level), and their ability to rely on children during serious problems increased by 0.10 units (3.0%) compared to the mean of 3.30 (significant at the 5% significant level). However, the program did not significantly affect children's understanding of respondents' feelings, which showed a marginal increase of 0.04 units compared to the mean value of 3.10. In Panel B, PLTCI implementation reduced the frequency of spouses criticizing respondents by 0.07 units, representing a 3.4% decrease relative to the mean value of 2.14 in control states without PLTCI (significant at the 5% significant level). Similarly, the frequency of spouses letting respondents down decreased by 0.08 units (3.8%) compared to the mean of 1.98 (significant at the 1% significant level). However, the program had no significant impact on the annoyance caused by spouses, with an insignificant decrease of 0.002 units relative to the mean of 1.77. Additionally, PLTCI did not significantly influence respondents' ability to open up about worries to their spouses, showing a small decrease of 0.02 units compared to the mean value of 3.44. The ability to rely on spouses during serious problems also remained unchanged, with a negligible increase of 0.01 units relative to the mean of 3.77. Similarly, spouses' understanding of respondents' feelings showed no significant improvement, with an increase of 0.03 units compared to the mean value of 3.31.

**TABLE 2:** The effect of the PLTCI implementation on relationship with children and spouse.

	(1)	(2)	(3)	(4)	(5)	(6)
	Criticize	Let You Down	Get on Nerves	Open Up Worries	Rely on Problems	Understand Feelings
Panel A: Children						
PLTCI implementation	−0.085**	−0.069**	−0.065**	0.065*	0.100**	0.036
	(0.040)	(0.027)	(0.028)	(0.039)	(0.037)	(0.027)
Mean of DV	1.994	1.762	1.846	3.038	3.295	3.103
Number of obs.	15,104	15,063	15,229	15,101	15,123	15,113
Panel B: Spouse						
PLTCI implementation	−0.072**	−0.075***	−0.002	−0.023	0.005	0.025
	(0.034)	(0.027)	(0.025)	(0.032)	(0.016)	(0.033)
Mean of DV	2.141	1.976	1.773	3.438	3.768	3.309
Number of obs.	13,230	13,215	13,232	13,238	13,245	13,226

*Note:* The table reports the effect of the Partnership Long‐Term Care Insurance (PLTCI) implementation on relationship with key network of respondents using the working sample of HRS individuals who are age eligible (no more than 65) and health eligible (no Activities of Daily Living [ADL] limitations) during the period in which the PLTCI program was in place, between 2004 and 2018. The specification in each column includes year and the PLTCI‐expansion‐group fixed effects. The mean of the dependent variables (DV) indicates the average of each variable in each column for the states that did not have PLTCI in place. Panel A shows the estimates on relationship with children for individuals that had at least one child in the study period. Panel B shows the estimates on relationship with spouses for individuals that were partnered or married in the study period. Each column represents a dependent variable measuring a specific aspect of respondents' relationships: *Criticize* (“How much do they criticize you?”), *Let You Down* (“How much do they let you down when you are counting on them?”), *Get on Nerves* (“How much do they get on your nerves?”), *Open Up Worries* (“How much can you open up to them if you need to talk about your worries?”), *Rely on Problems* (“How much can you rely on them if you have a serious problem?”), and *Understand Feelings* (“How much do they really understand the way you feel about things?”). Responses are on a four‐point Likert scale from 1 (“Not at all”), 2 (“A little”), 3 (“Some”), and 4 (“A lot”). Standard errors are clustered at the state level.

* *p* < 0.10.

** *p* < 0.05.

*** *p* < 0.01.

As robustness checks to the main results in Table 2, we employ two alternative specifications: one incorporating state and year fixed effects (Supporting Information: Table A2), and another using a recently developed approach for staggered policy adoption settings [30] (Supporting Information: Table A3). Both approaches yield results that are largely consistent with those reported in Table 2.

Supporting Information: Table A4 presents the estimated effects of PLTCI implementation on respondents' relationships with friends, revealing no significant changes across most dimensions. The program did not significantly affect the frequency of friends criticizing respondents, showing a negligible decrease of 0.004 units. Similarly, no significant change was observed in the frequency of friends letting respondents down, with a slight increase of 0.01 units (0.5%) compared to the mean of 1.50. However, the annoyance caused by friends decreased significantly by 0.05 units (2.9%) relative to the mean value of 1.59. In terms of positive aspects of the relationship, the program had no significant impact on respondents' ability to open up about worries to friends, nor did it significantly affect their ability to rely on friends during serious problems. Similarly, friends' understanding of respondents' feelings remained largely unchanged, with only a marginal increase of 0.01 units compared to the mean value of 3.12.

### 3.1 Heterogeneous Results

The main results may mask important heterogeneous effects across different groups. In this section, we present how PLTCI implementation affected relationships with key network members—children and spouses—across four dimensions: age, gender, race/ethnicity, and household wealth of individuals.

**FIGURE 1:**
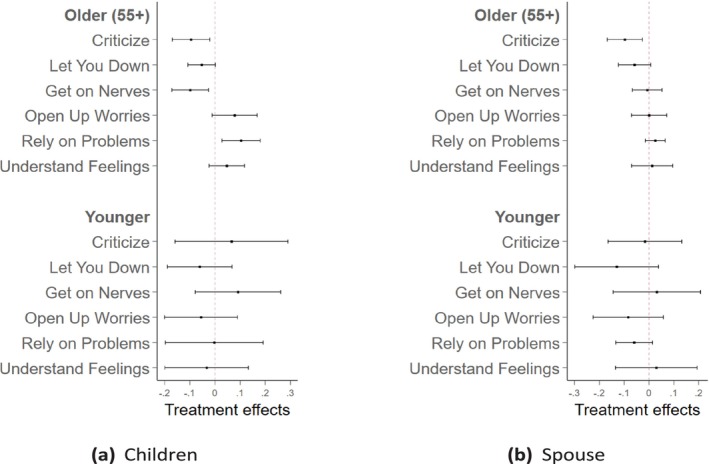
The heterogeneous effects of PLTCI implementation on the relationship with children and spouse by age. The graphs draw the heterogeneous effect with 95% confidence intervals of the Partnership Long‐Term Care Insurance (PLTCI) implementation on respondents' relationships with their children in plot (a) and spouses in plot (b) by age, using the working sample of HRS individuals who are age eligible (no more than 65) and health eligible (no Activities of Daily Living [ADL] limitations) during the period in which the PLTCI program was in place, between 2004 and 2018. The relatively older group includes individuals who are 55 and above, and the younger group includes individuals who are below 55. The model adjusts for year fixed effects and PLTCI‐expansion‐group fixed effects.

Figure 1 illustrates the heterogeneous effects of PLTCI on relationship improvements, with panel (a) focusing on individuals' relationships with their children and panel (b) on their relationships with their spouses. For the relatively older group, comprising individuals aged 55 and above, the treatment effects of PLTCI show significant improvements in their relationships with their children (Panel (a)). Perceptions of their children being critical, disappointing, or annoying were significantly reduced. In terms of positive relational dimensions, these individuals more frequently shared their worries with their children, felt more confident relying on them for serious issues, and perceived their children as better understanding their emotions. In comparison, for younger individuals, there were no significant effects across any dimensions, with large confidence intervals likely due to the smaller sample size. In Panel (b), which focused on relationship improvements with individuals' spouses, a similar pattern emerged. The improvement was primarily reflected in a reduction in the frequency of spouses criticizing or letting individuals down in the older group. Supporting Information: Tables A5 and A6 present the detailed coefficients for these dimensions in the outcomes of relationships with children and spouses, respectively.

**FIGURE 2:**
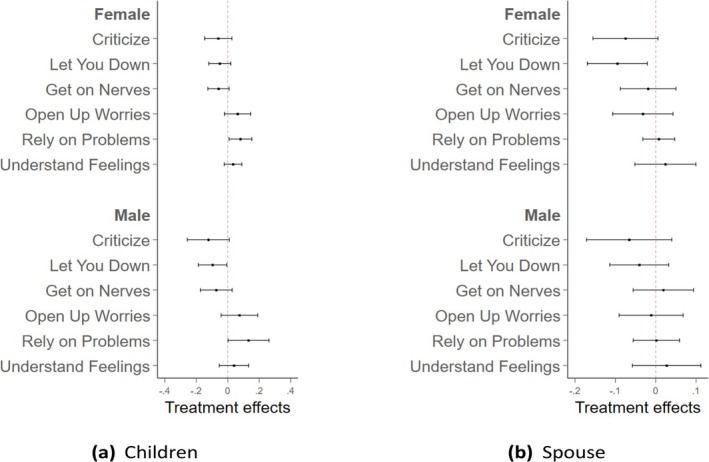
The heterogeneous effects of PLTCI implementation on the relationship with children and spouse by gender. The graphs draw the heterogeneous effect with 95% confidence intervals of the Partnership Long‐Term Care Insurance (PLTCI) implementation on respondents' relationships with their children in plot (a) and spouses in plot (b) by gender, using the working sample of HRS individuals who are age eligible (no more than 65) and health eligible (no Activities of Daily Living [ADL] limitations) during the period in which the PLTCI program was in place, between 2004 and 2018. The model adjusts for year fixed effects and PLTCI‐expansion‐group fixed effects.

Figure 2 further illustrates the heterogeneous patterns by gender. As shown in Panel (a), which focuses on relationships with children, PLTCI improves the quality of these relationships across various dimensions for both women and men. In general, individuals experienced less criticism, disappointment, or annoyance, and more frequent sharing of worries, greater reliance on children when problems arose, and more instances of feeling understood. For relationships with spouses (Panel (b)), the improvement was more noticeable in the dimensions of reduced criticism and fewer instances of being let down; but this was observed only in women. Men reported fewer relationship improvements overall. Supporting Information: Tables A7 and A8 present the detailed coefficients for these dimensions in the outcomes of relationships with children and spouses, respectively.

**FIGURE 3:**
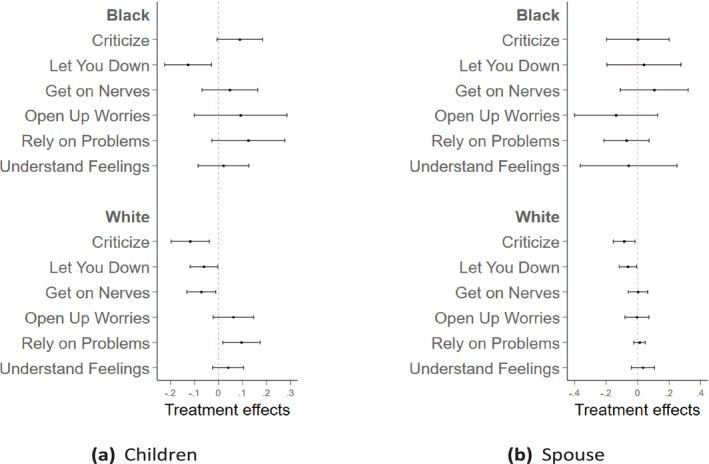
The heterogeneous effects of PLTCI implementation on the relationship with children and spouse by race and ethnicity. The graphs draw the heterogeneous effect with 95% confidence intervals of the Partnership Long‐Term Care Insurance (PLTCI) implementation on respondents' relationships with their children in plot (a) and spouses in plot (b) by race, using the working sample of HRS individuals who are age eligible (no more than 65) and health eligible (no Activities of Daily Living [ADL] limitations) during the period in which the PLTCI program was in place, between 2004 and 2018. The model adjusts for year fixed effects and PLTCI‐expansion‐group fixed effects.

Additionally, Figure 3 presents the heterogeneous effects by race and ethnicity. Due to the small sample sizes of Hispanic and non‐Hispanic individuals of other races in the non‐PLTCI states, we excluded these groups from the heterogeneous analysis. Only non‐Hispanic White (White) and non‐Hispanic Black (Black) individuals are included in this sample. For relationships with children, as shown in Panel (a), significant improvements were observed only for White individuals. These improvements included statistically significant reductions in negative experiences, such as less criticism, disappointment, and annoyance from their children. On the positive side, reliance on children for serious problems also improved significantly. In contrast, for Black individuals, the relational effects are mixed, with evidence of less disappointment but more criticism. For other dimensions, the effects are not statistically significant. For spousal relationships, as shown in Panel (b), White individuals experienced reductions in negative feelings toward their spouses, particularly with significant decreases in criticism and disappointment. However, for Black individuals, the effects were minimal. If anything, positive relational experiences, such as sharing worries and relying on spouses for support, appeared to deteriorate slightly. Supporting Information: Tables A9 and A10 present the detailed coefficients for these dimensions in the outcomes of relationships with children and spouses, respectively.

**FIGURE 4:**
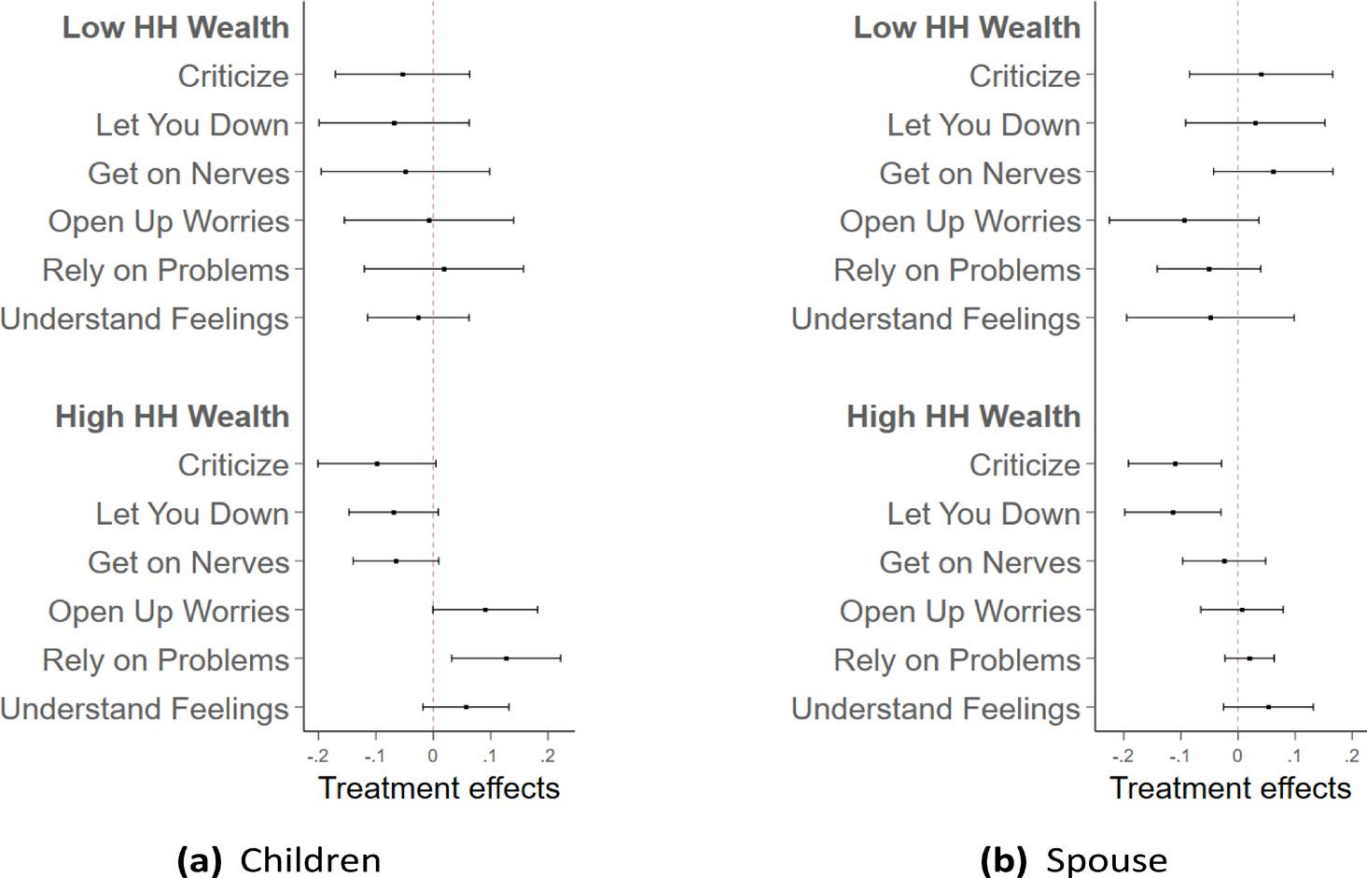
The heterogeneous effects of PLTCI implementation on the relationship with children and spouse by household wealth. The graphs draw the heterogeneous effect with 95% confidence intervals of the Partnership Long‐Term Care Insurance (PLTCI) implementation on respondents' relationships with their children in plot (a) and spouses in plot (b) by household wealth, using the working sample of HRS individuals who are age eligible (no more than 65) and health eligible (no Activities of Daily Living [ADL] limitations) during the period in which the PLTCI program was in place, between 2004 and 2018. Low household wealth means that the total wealth is below the 50th percentile (approx. $169,000) of the distribution in the study period in the working sample. The total wealth is defined as the net value of household wealth calculated by subtracting the value of all liabilities from the value of all assets owned by the household. The model adjusts for year fixed effects and PLTCI‐expansion‐group fixed effects.

We also examine the patterns by household wealth, as shown in Figure 4. For individuals with higher household wealth (above the median), PLTCI implementation significantly improved the quality of their relationships with their children. These improvements included fewer negative feelings toward their children and more frequent positive feelings from them, as illustrated in Panel (a). In contrast, for individuals with lower household wealth, these improvements were less evident, with estimates across all relational dimensions being statistically insignificant. For spousal relationships, as shown in Panel (b), relatively wealthier individuals experienced significant reductions in criticism and disappointment from their spouses, although effects on other dimensions were statistically negligible. Among individuals with lower household wealth, improvements were not observed across all dimensions of spousal relationships, and these effects lacked statistical significance. Supporting Information: Tables A11 and A12 present the detailed coefficients for these dimensions in the outcomes of relationships with children and spouses, respectively.

Finally, it is informative to examine the heterogeneous effects of PLTCI based on LTCI ownership. Supporting Information: Tables A13 and A14 present these results for relationships with children and spouses, respectively. Among individuals with LTCI, the reduction in negative feelings—such as criticism and disappointment—toward their spouse is significantly greater, showing a 1.8‐unit decrease (significant at the 5% level), compared to a near‐zero effect among those without LTCI. For relationships with children, individuals with LTCI also show more pronounced improvements, including a 2.4‐unit increase in feeling comfortable opening up about worries, a 2.6‐unit increase in relying on problem‐solving, and a 2.3‐unit increase in feeling understood. These effects are substantially larger than those observed among individuals without LTCI, where the corresponding improvements are just 0.04, 0.07, and 0.10 units, respectively.

## 4 Discussion

The paper provides evidence that PLTCI program that encourages LTCI purchase can enhance relationship quality between potential eligible individuals and their adult children or spouses, particularly improving intergenerational relationships. Utilizing a larger, representative survey dataset of older Americans, the study finds that the availability of PLTCI program reduces negative emotions, such as criticism, frustration, and irritation, while fostering more positive interactions, including greater emotional support, shared concerns, and improved problem communication with adult children and spouses. Despite the significant implications of LTC policies, direct evidence on how general LTC programs influence personal relationships remains scarce in the literature. The findings of this study suggest potential mechanisms through which LTCI or LTC subsidy programs contribute to better health outcomes and overall well‐being for aging individuals or caregivers [11, 12, 14, 15, 21, 31]. Additionally, these findings help explain why the availability of LTCI can alleviate strain in parent–child relationships. Existing research highlights two key perspectives. One branch of studies explores how access to informal care from family members reduces the demand for LTCI [32, 33]. Another branch documents the presence of ambivalence in close relationships, particularly in the context of caregiving [34, 35, 36, 37]. For instance, it is demonstrated that adult children caring for parents with intensive needs such as those requiring assistance with ADLs, often experience significant burdens and heightened negative emotions [34]. In contrast, when parents require short‐term care, such as recovering from a fall or a broken hip, the emotional effects can sometimes be positive. Similarly, while providing support to grown children is generally associated with positive emotions, offering support to aging parents tends to elicit more negative moods [36]. Middle‐aged adults, in particular, experience complex emotions due to their involvement with aging parents. These findings underscore the ambivalent nature of relationships within key social networks. The close‐but‐distant dynamic often leads to conflicts arising from various factors, including LTC needs, caregiving responsibilities, and financial support. LTCI coverage can significantly mitigate these tensions by reducing the need for intensive family discussions, alleviating caregiving burdens on family members, and improving financial security through LTC subsidies. Collectively, these benefits contribute to an overall enhancement in relationship quality.

In addition, the findings contribute to the existing literature by proposing potential interventions to enhance the social network quality. Beyond psychosocial therapy interventions designed to strengthen social connections [16], various approaches have been explored to foster meaningful relationships and improve social well‐being among aging adults [17, 18]. These include initiatives that encourage intergenerational interactions, the development of aging‐friendly communities, and community‐based programs that promote social engagement and physical activity. Moreover, advancements in technology have provided new opportunities to maintain communication and strengthen bonds with family members and peers. While these interventions have shown promise in mitigating social isolation and enhancing emotional well‐being, the expansion of the PLTCI program presents an additional avenue for improving relationship quality. By alleviating caregiving‐related tensions, reducing financial stress, and ensuring access to necessary LTC services, LTCI can play a crucial role in fostering more positive and supportive relationships within individuals' social networks.

Expanding on these findings, policy initiatives should prioritize integrating social support strategies within LTCI frameworks to maximize their impact on social well‐being. Policymakers could consider incorporating provisions that facilitate access to community‐based social programs as part of LTCI benefits, ensuring that enrollees receive not only medical and caregiving support but also opportunities for meaningful social engagement. Additionally, regulatory frameworks could incentivize LTCI providers to collaborate with local organizations to offer social activities, digital literacy training, and transportation services that enhance social connectivity. By embedding these elements into LTCI policies, governments and stakeholders can create a more holistic support system that not only addresses the practical aspects of aging but also actively fosters stronger, healthier social networks among aging individuals.

### 4.1 Limitations

This paper aims to provide evidence on how the PLTCI program may improve the well‐being of aging adults by enhancing their relationships within key social networks. First, it is important to note that the estimates presented here are intent‐to‐treat estimates rather than average treatment‐on‐treated estimates, as data on actual LTCI purchases under the PLTCI policy in HRS is unavailable. Additionally, for those eligible but not enrolled in LTCI, we lack insight into unmeasured factors that could account for their rejection from LTCI coverage. Second, the subsample of survey respondents who completed the questionnaire may not be fully representative of the target population, as some individuals may have opted out of completing the survey or declined to respond.

To assess the extent of this limitation, we conducted the following checks. First, we compared the summary statistics of the leave‐behind working sample with those of the full HRS sample to determine whether there are notable differences between the two and to evaluate how representative the working sample is (Supporting Information: Table A15). Second, assuming the leave‐behind sample is representative, we applied the core HRS weights to our analysis and found that the results remained largely unchanged (Supporting Information: Table A17). Third, we used a specifically designed weight tailored for the leave‐behind subsample and re‐estimated the main effects of the PLTCI program on relational outcomes with children and spouses. The results remained highly consistent (Supporting Information: Table A16). Overall, these checks suggest that the leave‐behind HRS subsample provides a reasonable representation of the broader aging population in the full HRS, and any potential nonresponse bias appears to be minimal. In addition, individuals who had no spouse or partner and/or no children were excluded from the analysis. As a result, the effects of the LTCI policy on the daily lives of this group remain unexamined. Future research could provide valuable insights into how the policy impacts this segment of the population. Third, while the leave‐behind questionnaire provides detailed information on respondents' social networks, it does not capture the full picture of how policy that encourages LTCI purchase might impact various aspects of aging adults' lives, including shifts in family dynamics, access to caregiving support, and the overall quality of care they receive.

## 5 Conclusion

In conclusion, this study aimed to understand what PLTCI program meant for the well‐being of aging adults, moving beyond prior research, which primarily examined its impact on formal and informal care. Unlike previous studies, this research focused on how the potential eligibility for PLTCI influenced the daily lives and relationships of aging individuals. Using data from the Participant Lifestyle Questionnaire of the Leave‐Behind Survey in the HRS, combined with the large‐scale implementation of the PLTCI policy, we found that PLTCI significantly strengthened relationships with key social networks of potential eligible HRS respondents. Specifically, respondents residing in states with PLTCI policies experienced fewer feelings of criticism, fewer frustrations over unmet communication needs, and fewer instances of feeling irritated by their network members. They were also more likely to confide in close connections, seek support during serious challenges, and feel understood by others. These positive effects on relationships were especially notable with children and spouses. Additionally, these improvements were most evident for non‐Hispanic White, higher‐wealth households were similar for both men and women.

The findings from this study suggest important policy implications for the role of LTCI in enhancing the social well‐being of aging adults. By improving relationships with key social networks, particularly with children and spouses, the PLTCI policy appears to foster stronger, more supportive connections, which could be crucial for the emotional and psychological well‐being of individuals. This highlights the potential benefits of expanding access to LTCI as a means to not only address care needs but also to enhance the quality of interpersonal relationships. Policymakers may consider further promoting and expanding such programs, particularly for individuals in higher‐wealth households or specific demographic groups, to reduce social isolation and strengthen family support networks. Additionally, the positive effects observed across both men and women suggest that LTCI programs have broad applicability, benefiting all genders and potentially reducing caregiving burdens within families.

## Conflicts of Interest

The author declares no conflicts of interest.

## Supporting information


**Data S1:** Supporting information.

## Data Availability

The data that support the findings of this study are available from University of Michigan. Restrictions apply to the availability of these data, which were used under license for this study. Data are available from https://hrsdata.isr.umich.edu/rda with the permission of University of Michigan.
