# Supplementary material for: From Criticism to Comfort: The Relational Benefits of Long‐Term Care Insurance
Source: Health Serv Res. 2025 Aug 13;61(1):e70026. doi: 10.1111/1475-6773.70026 (PMC12857447; doi:10.1111/1475-6773.70026)
Supplement: Supplementary file 1 — Data S1: Supporting information. [file HESR-61-e70026-s001.docx]

Appendix Tables

# Table A1: Year of the PLTCI Implementation by State

| State | Implementation Year | State | Implementation Year |
| --- | --- | --- | --- |
| Alabama | 2009 | Montana | 2009 |
| Alaska | No PLTCI | Nebraska | 2006 |
| Arizona | 2008 | Nevada | 2007 |
| Arkansas | 2008 | New Hampshire | 2010 |
| California | Pilot State | New Jersey | 2008 |
| Colorado | 2008 | New Mexico | 2019 |
| Connecticut | Pilot State | New York | Pilot |
| Delaware | 2011 | North Carolina | 2011 |
| Florida | 2007 | North Dakota | 2007 |
| Georgia | 2007 | Ohio | 2007 |
| Hawaii | No PLTCI | Oklahoma | 2008 |
| Idaho | 2006 | Oregon | 2008 |
| Illinois | 2019 | Pennsylvania | 2007 |
| Indiana | Pilot State | Rhode Island | 2008 |
| Iowa | 2010 | South Carolina | 2009 |
| Kansas | 2007 | South Dakota | 2007 |
| Kentucky | 2008 | Tennessee | 2008 |
| Louisiana | 2009 | Texas | 2008 |
| Maine | 2009 | Utah | No PLTCI |
| Maryland | 2009 | Vermont | 2020 |
| Massachusetts | 2020 | Virginia | 2007 |
| Michigan | 2016 | Washington | 2012 |
| Minnesota | 2006 | West Virginia | 2010 |
| Mississippi | No PLTCI | Wisconsin | 2009 |
| Missouri | 2008 | Wyoming | 2009 |

*Note*. States implemented the Partnership Long-Term Care Insurance (PLTCI) program in the study period 2004-2018 in the same year as follows: 2006 (3 states: Nebraska, Idaho, Minnesota), 2007 (9 states: Nevada, Florida, Georgia, North Dakota, Ohio, Pennsylvania, South Dakota, Virginia, Kansas), 2008 (11 states: Arizona, Arkansas, New Jersey, Colorado, Oklahoma, Oregon, Rhode Island, Kentucky, Tennessee, Texas, Missouri), 2009 (8 states: Alabama, Montana, South Carolina, Louisiana, Maine, Maryland, Wisconsin, Wyoming), 2010 (3 states: New Hampshire, Iowa, West Virginia), 2011 (2 states: Delaware, North Carolina), 2012 (1 state: Washington), 2016 (1 state: Michigan), California, Connecticut, Indiana, and New York are designated as Pilot States. Alaska, Hawaii, Mississippi, and Utah have no PLTCI program.

# Table A2: Robustness Check: The Effect of the PLTCI Implementation on Relationship with Children and Spouse

|  | (1) | (2) | (3) | (4) | (5) | (6) |
| --- | --- | --- | --- | --- | --- | --- |
|  |  |  |  | Panel A: Children |  |  |
|  | Criticize | Let You Down | Get on Nerves | Open Up Worries | Rely on Problems | Understand Feelings |
| PLTCI implementation | -0.084** | -0.066** | -0.063** | 0.064 | 0.094** | 0.032 |
|  | (0.040) | (0.027) | (0.028) | (0.039) | (0.038) | (0.028) |
| Mean of DV. | 1.994 | 1.762 | 1.846 | 3.038 | 3.295 | 3.103 |
| Number of Obs. | 15,103 | 15,063 | 15,229 | 15,101 | 15,123 | 15,113 |
|  |  |  |  | Panel B: Spouse |  |  |
|  | Criticize | Let You Down | Get on Nerves | Open Up Worries | Rely on Problems | Understand Feelings |
| PLTCI implementation | -0.068** | -0.067** | 0.003 | -0.026 | 0.000 | 0.020 |
|  | (0.034) | (0.026) | (0.025) | (0.032) | (0.014) | (0.033) |
| Mean of DV. | 2.141 | 1.976 | 1.773 | 3.438 | 3.768 | 3.309 |
| Number of Obs. | 13,229 | 13,214 | 13,231 | 13,237 | 13,244 | 13,225 |

*Note*. The table reports the robustness check of the effect of the Partnership Long-Term Care Insurance (PLTCI) implementation on relationship with key network of respondents using the working sample of HRS individuals who are age eligible (no more than 65) and health eligible (no Activities of Daily Living (ADL) limitations) during the period in which the PLTCI program was in place, between 2004 and 2018. The alternative specification in each column includes year and state fixed effects. The mean of the dependent variables (DV.) indicates the average of each variable in each column for the states that did not have PLTCI in place. Panel A shows the estimates on relationship with children for individuals that had at least one child in the study period. Panel B shows the estimates on relationship with spouses for individuals that were partnered or married in the study period. Each column represents a dependent variable measuring a specific aspect of respondents’ relationships: *Criticize* (“How much do they criticize you?”), *Let You Down* (“How much do they let you down when you are counting on them?”), *Get on Nerves* (“How much do they get on your nerves?”), *Open Up Worries* (“How much can you open up to them if you need to talk about your worries?”), *Rely on Problems* (“How much can you rely on them if you have a serious problem?”) and *Understand Feelings* (“How much do they really understand the way you feel about things?”). Responses are on a four-point Likert scale from 1 (“Not at all”), 2 (“A little”), 3 (“Some”), and 4 (“A lot”). Standard errors are clustered at the state level. *** p*<*0.01, ** p*<*0.05, * p*<*0.10.

# Table A3: Robustness Check: The Effect of the PLTCI Implementation on Relationship with Children and Spouse Using BJS Method

|  | (1) | (2) | (3) | (4) | (5) | (6) |
| --- | --- | --- | --- | --- | --- | --- |
| Panel A: Children | | | | | | |
|  | Criticize | Let You Down | Get on Nerves | Open Up Worries | Rely on Problems | Understand Feelings |
| PLTCI implementation | -0.137*** | -0.0822*** | -0.118*** | 0.0736*** | 0.131*** | 0.0881*** |
|  | (0.0289) | (0.0262) | (0.0266) | (0.0265) | (0.0184) | (0.0260) |
| Mean of DV. | 1.994 | 1.762 | 1.846 | 3.038 | 3.295 | 3.103 |
| Number of Obs. | 10,620 | 10,590 | 10,618 | 10,622 | 10,640 | 10,633 |
|  |  |  |  | Panel B: Spouse |  |  |
|  | Criticize | Let You Down | Get on Nerves | Open Up Worries | Rely on Problems | Understand Feelings |
| PLTCI implementation | -0.149*** | -0.0580** | -0.0190 | 0.00983 | -0.0210 | 0.0755*** |
|  | (0.0253) | (0.0278) | (0.0141) | (0.0243) | (0.0167) | (0.0218) |
| Mean of DV. | 2.141 | 1.976 | 1.773 | 3.438 | 3.768 | 3.309 |
| Number of Obs. | 9,313 | 9,301 | 9,318 | 9,324 | 9,329 | 9,311 |

*Note*. The table reports the robustness check of the effect of the Partnership Long-Term Care Insurance (PLTCI) implementation on relationship with key network of respondents using the working sample of HRS individuals who are age eligible (no more than 65) and health eligible (no Activities of Daily Living (ADL) limitations) during the period in which the PLTCI program was in place, between 2004 and 2018. The alternative specification in each column uses the approach proposed by Borusyak et al. (2024). The mean of the dependent variables (DV.) indicates the average of each variable in each column for the states that did not have PLTCI in place. Panel A shows the estimates on relationship with children for individuals that had at least one child in the study period. Panel B shows the estimates on relationship with spouses for individuals that were partnered or married in the study period. Each column represents a dependent variable measuring a specific aspect of respondents’ relationships: *Criticize* (“How much do they criticize you?”), *Let You Down* (“How much do they let you down when you are counting on them?”), *Get on Nerves* (“How much do they get on your nerves?”), *Open Up Worries* (“How much can you open up to them if you need to talk about your worries?”), *Rely on Problems* (“How much can you rely on them if you have a serious problem?”) and *Understand Feelings* (“How much do they really understand the way you feel about things?”). Responses are on a four-point Likert scale from 1 (“Not at all”), 2 (“A little”), 3 (“Some”), and 4 (“A lot”). Standard errors are clustered at the state level. *** p*<*0.01, ** p*<*0.05, * p*<*0.10.

# Table A4: The Effect of the PLTCI Implementation on Relationship with Friends

|  | Criticize | Let You Down | Get on Nerves | Open Up Worries | Rely on Problems | Understand Feelings |
| --- | --- | --- | --- | --- | --- | --- |
|  | (1) | (2) | (3) | (4) | (5) | (6) |
| PLTCI implementation | -0.004 | 0.007 | -0.046* | -0.036 | 0.036 | 0.012 |
|  | (0.020) | (0.022) | (0.026) | (0.032) | (0.037) | (0.030) |
| Mean of DV. | 1.455 | 1.503 | 1.591 | 3.105 | 3.144 | 3.115 |
| Number of Obs. | 16,474 | 16,403 | 16,459 | 16,495 | 16,503 | 16,502 |

*Note*. The table reports the effect of the Partnership Long-Term Care Insurance (PLTCI) implementation on respondents’ relationships with their friends using the working sample of HRS individuals who are age eligible (no more than 65) and health eligible (no Activities of Daily Living (ADL) limitations) during the period in which the program was in place, between 2004 and 2018. The specification in each column includes year and the PLTCI-expansion-group fixed effects. The mean of the dependent variables (DV.) indicates the average of each variable in each column for the states that did not have PLTCI in place. Each column represents a dependent variable measuring a specific aspect of respondents’ relationships with their respective group: *Criticize* (“How much do they criticize you?”), *Let You Down* (“How much do they let you down when you are counting on them?”), *Get on Nerves* (“How much do they get on your nerves?”), *Open Up Worries* (“How much can you open up to them if you need to talk about your worries?”), *Rely on Problems* (“How much can you rely on them if you have a serious problem?”) and *Understand Feelings* (“How much do they really understand the way you feel about things?”). Responses are on a four-point Likert scale from 1 (“Not at all”), 2 (“A little”), 3 (“Some”), and 4 (“A lot”). Standard errors are clustered at the state level.

*** p*<*0.01, ** p*<*0.05, * p*<*0.10.

# Table A5: The Heterogeneous Effects of PLTCI Implementation on the Relationship with Children by Age

|  | (1) | (2) | (3) | (4) | (5) | (6) |
| --- | --- | --- | --- | --- | --- | --- |
|  |  |  |  | Panel A: Older (55+) |  |  |
|  | Criticize | Let You Down | Get on Nerves | Open Up Worries | Rely on Problems | Understand Feelings |
| PLTCI implementation | -0.095** | -0.053* | -0.099*** | 0.078* | 0.104*** | 0.047 |
|  | (0.037) | (0.027) | (0.036) | (0.044) | (0.038) | (0.035) |
| Mean of DV. | 1.917 | 1.698 | 1.823 | 3.072 | 3.344 | 3.119 |
| Number of Obs. | 11,812 | 11,778 | 11,926 | 11,801 | 11,821 | 11,814 |
|  |  |  |  | Panel B: Younger |  |  |
|  | Criticize | Let You Down | Get on Nerves | Open Up Worries | Rely on Problems | Understand Feelings |
| PLTCI implementation | 0.066 | -0.061 | 0.091 | -0.056 | -0.003 | -0.033 |
|  | (0.111) | (0.063) | (0.084) | (0.072) | (0.096) | (0.082) |
| Mean of DV. | 2.119 | 1.866 | 1.883 | 2.984 | 3.217 | 3.076 |
| Number of Obs. | 3,290 | 3,283 | 3,301 | 3,298 | 3,300 | 3,297 |

*Notes*. The table reports the heterogeneous effects of the Partnership Long-Term Care Insurance (PLTCI) implementation on relationships with the children of respondents by age, using the working sample of HRS individuals who are age eligible (no more than 65) and health eligible (no Activities of Daily Living (ADL) limitations) during the period in which the program was in place, between 2004 and 2018. The specification in each column includes year and the PLTCI-expansion-group fixed effects. Panel A shows the older group which includes individuals who are above 55 and below 65 and Panel B depicts the younger group which includes individuals who are below 55. The mean of the dependent variables (DV.) indicates the average of each variable in each column for the states that did not have PLTCI in place. Each column represents a dependent variable measuring a specific aspect of respondents’ relationships: *Criticize* (“How much do they criticize you?”), *Let You Down* (“How much do they let you down when you are counting on them?”), *Get on Nerves* (“How much do they get on your nerves?”), *Open Up Worries* (“How much can you open up to them if you need to talk about your worries?”), *Rely on Problems* (“How much can you rely on them if you have a serious problem?”) and *Understand Feelings* (“How much do they really understand the way you feel about things?”). Responses are on a four-point Likert scale from 1 (“Not at all”), 2 (“A little”), 3 (“Some”), and 4 (“A lot”). Standard errors are clustered at the state level.

*** p*<*0.01, ** p*<*0.05, * p*<*0.10.

# Table A6: The Heterogeneous Effects of PLTCI Implementation on the Relationship with Spouse by Age

|  | (1) | (2) | (3) | (4) | (5) | (6) |
| --- | --- | --- | --- | --- | --- | --- |
| Panel A: Older (55+) | | | | | | |
|  | Criticize | Let You Down | Get on Nerves | Open Up Worries | Rely on Problems | Understand Feelings |
| PLTCI implementation | -0.097*** | -0.058* | -0.007 | 0.000 | 0.025 | 0.012 |
|  | (0.035) | (0.032) | (0.030) | (0.035) | (0.020) | (0.041) |
| Mean of DV. | 2.157 | 2.014 | 1.736 | 3.420 | 3.764 | 3.326 |
| Number of Obs. | 9,927 | 9,919 | 9,929 | 9,938 | 9,943 | 9,928 |
|  |  |  |  | Panel B: Younger |  |  |
|  | Criticize | Let You Down | Get on Nerves | Open Up Worries | Rely on Problems | Understand Feelings |
| PLTCI implementation | -0.017 | -0.130 | 0.031 | -0.084 | -0.060 | 0.029 |
|  | (0.073) | (0.083) | (0.087) | (0.070) | (0.036) | (0.081) |
| Mean of DV. | 2.119 | 1.922 | 1.826 | 3.464 | 3.774 | 3.284 |
| Number of Obs. | 3,303 | 3,296 | 3,303 | 3,300 | 3,302 | 3,298 |

*Notes*. The table reports the heterogeneous effects of the Partnership Long-Term Care Insurance (PLTCI) implementation on relationships with the spouse of respondents by age, using the working sample of HRS individuals who are age eligible (no more than 65) and health eligible (no Activities of Daily Living (ADL) limitations) during the period in which the program was in place, between 2004 and 2018. The specification in each column includes year and the PLTCI-expansion-group fixed effects. Panel A shows the older group which includes individuals who are above 55 and below 65 and Panel B depicts the younger group which includes individuals who are below 55. The mean of the dependent variables (DV.) indicates the average of each variable in each column for the states that did not have PLTCI in place. Each column represents a dependent variable measuring a specific aspect of respondents’ relationships: *Criticize* (“How much do they criticize you?”), *Let You Down* (“How much do they let you down when you are counting on them?”), *Get on Nerves* (“How much do they get on your nerves?”), *Open Up Worries* (“How much can you open up to them if you need to talk about your worries?”), *Rely on Problems* (“How much can you rely on them if you have a serious problem?”) and *Understand Feelings* (“How much do they really understand the way you feel about things?”). Responses are on a four-point Likert scale from 1 (“Not at all”), 2 (“A little”), 3 (“Some”), and 4 (“A lot”). Standard errors are clustered at the state level.

*** p*<*0.01, ** p*<*0.05, * p*<*0.10.

# Table A7: The Heterogeneous Effects of PLTCI Implementation on the Relationship with Children by Gender

|  | (1) | (2) | (3) | (4) | (5) | (6) |
| --- | --- | --- | --- | --- | --- | --- |
|  |  |  |  | Panel A: Female |  |  |
|  | Criticize | Let You Down | Get on Nerves | Open Up Worries | Rely on Problems | Understand Feelings |
| PLTCI implementation | -0.060 | -0.051 | -0.059* | 0.062 | 0.080** | 0.034 |
|  | (0.043) | (0.034) | (0.033) | (0.041) | (0.036) | (0.028) |
| Mean of DV. | 2.013 | 1.773 | 1.849 | 3.163 | 3.369 | 3.177 |
| Number of Obs. | 9,130 | 9,095 | 9,221 | 9,123 | 9,142 | 9,134 |
|  |  |  |  | Panel B: Male |  |  |
|  | Criticize | Let You Down | Get on Nerves | Open Up Worries | Rely on Problems | Understand Feelings |
| PLTCI implementation | -0.123* | -0.097** | -0.073 | 0.074 | 0.132** | 0.039 |
|  | (0.066) | (0.045) | (0.050) | (0.057) | (0.064) | (0.046) |
| Mean of DV. | 1.962 | 1.745 | 1.841 | 2.830 | 3.171 | 2.980 |
| Number of Obs. | 5,974 | 5,968 | 6,008 | 5,978 | 5,981 | 5,979 |

*Notes*. The table reports the heterogeneous effects of the Partnership Long-Term Care Insurance (PLTCI) implementation on relationships with the children of respondents by gender, using the working sample of HRS individuals who are age eligible (no more than 65) and health eligible (no Activities of Daily Living (ADL) limitations) during the period in which the program was in place, between 2004 and 2018. The specification in each column includes year and the PLTCI-expansion-group fixed effects. The mean of the dependent variables (DV.) indicates the average of each variable in each column for the states that did not have PLTCI in place. Each column represents a dependent variable measuring a specific aspect of respondents’ relationships: *Criticize* (“How much do they criticize you?”), *Let You Down* (“How much do they let you down when you are counting on them?”), *Get on Nerves* (“How much do they get on your nerves?”), *Open Up Worries* (“How much can you open up to them if you need to talk about your worries?”), *Rely on Problems* (“How much can you rely on them if you have a serious problem?”) and *Understand Feelings* (“How much do they really understand the way you feel about things?”). Responses are on a four-point Likert scale from 1 (“Not at all”) to 4 (“A lot”). Standard errors are clustered at the state level.

*** p*<*0.01, ** p*<*0.05, * p*<*0.10.

# Table A8: The Heterogeneous Effects of PLTCI Implementation on the Relationship with Spouse by Gender

|  | (1) | (2) | (3) | (4) | (5) | (6) |
| --- | --- | --- | --- | --- | --- | --- |
|  |  |  |  | Panel A: Female |  |  |
|  | Criticize | Let You Down | Get on Nerves | Open Up Worries | Rely on Problems | Understand Feelings |
| PLTCI implementation | -0.075* | -0.095** | -0.019 | -0.032 | 0.008 | 0.024 |
|  | (0.040) | (0.037) | (0.034) | (0.037) | (0.020) | (0.037) |
| Mean of DV. | 2.134 | 1.987 | 1.886 | 3.372 | 3.720 | 3.216 |
| Number of Obs. | 7,398 | 7,391 | 7,400 | 7,404 | 7,409 | 7,392 |
|  |  |  |  | Panel B: Male |  |  |
|  | Criticize | Let You Down | Get on Nerves | Open Up Worries | Rely on Problems | Understand Feelings |
| PLTCI implementation | -0.066 | -0.041 | 0.019 | -0.011 | 0.001 | 0.027 |
|  | (0.052) | (0.036) | (0.037) | (0.039) | (0.028) | (0.042) |
| Mean of DV. | 2.151 | 1.960 | 1.613 | 3.534 | 3.837 | 3.440 |
| Number of Obs. | 5,832 | 5,824 | 5,832 | 5,834 | 5,836 | 5,834 |

*Notes*. The table reports the heterogeneous effects of the Partnership Long-Term Care Insurance (PLTCI) implementation on relationships with the spouse of respondents by gender, using the working sample of HRS individuals who are age eligible (no more than 65) and health eligible (no Activities of Daily Living (ADL) limitations) during the period in which the program was in place, between 2004 and 2018. The specification in each column includes year and the PLTCI-expansion-group fixed effects. The mean of the dependent variables (DV.) indicates the average of each variable in each column for the states that did not have PLTCI in place. Each column represents a dependent variable measuring a specific aspect of respondents’ relationships: *Criticize* (“How much do they criticize you?”), *Let You Down* (“How much do they let you down when you are counting on them?”), *Get on Nerves* (“How much do they get on your nerves?”), *Open Up Worries* (“How much can you open up to them if you need to talk about your worries?”), *Rely on Problems* (“How much can you rely on them if you have a serious problem?”) and *Understand Feelings* (“How much do they really understand the way you feel about things?”). Responses are on a four-point Likert scale from 1 (“Not at all”), 2 (“A little”), 3 (“Some”), and 4 (“A lot”). Standard errors are clustered at the state level.

*** p*<*0.01, ** p*<*0.05, * p*<*0.10.

# Table A9: The Heterogeneous Effects of PLTCI Implementation on the Relationship with Children by Race and Ethnicity

|  | (1) | (2) | (3) | (4) | (5) | (6) |
| --- | --- | --- | --- | --- | --- | --- |
| Panel A: Non-Hispanic Black | | | | | | |
|  | Criticize | Let You Down | Get on Nerves | Open Up Worries | Rely on Problems | Understand Feelings |
| PLTCI implementation | 0.089* | -0.127** | 0.047 | 0.092 | 0.125 | 0.021 |
|  | (0.046) | (0.048) | (0.057) | (0.095) | (0.075) | (0.052) |
| Mean of DV. | 1.924 | 1.760 | 1.766 | 3.206 | 3.270 | 3.231 |
| Number of Obs. | 2,366 | 2,355 | 2,400 | 2,360 | 2,372 | 2,370 |
| Panel B: Non-Hispanic White | | | | | | |
|  | Criticize | Let You Down | Get on Nerves | Open Up Worries | Rely on Problems | Understand Feelings |
| PLTCI implementation | -0.118*** | -0.061** | -0.072** | 0.062 | 0.096** | 0.040 |
|  | (0.039) | (0.028) | (0.030) | (0.042) | (0.039) | (0.032) |
| Mean of DV. | 1.998 | 1.760 | 1.854 | 3.012 | 3.308 | 3.080 |
| Number of Obs. | 11,241 | 11,219 | 11,322 | 11,244 | 11,250 | 11,244 |

*Notes*. The table reports the heterogeneous effects of the Partnership Long-Term Care Insurance (PLTCI) implementation on relationships with the children of respondents by race and ethnicity, using the working sample of HRS individuals who are age eligible (no more than 65) and health eligible (no Activities of Daily Living (ADL) limitations) during the period in which the program was in place, between 2004 and 2018.

Due to the small sample sizes of Hispanic and non-Hispanic individuals of other races in the non-PLTCI states, we excluded these groups from the heterogeneous analysis. Only non-Hispanic White (White) and non-Hispanic Black (Black) individuals are included in this sample.

The specification in each column includes year and the PLTCI-expansion-group fixed effects. The mean of the dependent variables (DV.) indicates the average of each variable in each column for the states that did not have PLTCI in place. Each column represents a dependent variable measuring a specific aspect of respondents’ relationships with their respective group: *Criticize* (“How much do they criticize you?”), *Let You Down* (“How much do they let you down when you are counting on them?”), *Get on Nerves* (“How much do they get on your nerves?”), *Open Up Worries* (“How much can you open up to them if you need to talk about your worries?”), *Rely on Problems* (“How much can you rely on them if you have a serious problem?”) and *Understand Feelings* (“How much do they really understand the way you feel about things?”). Responses are on a four-point Likert scale from 1 (“Not at all”), 2 (“A little”), 3 (“Some”), and 4 (“A lot”). Standard errors are clustered at the state level. *** p*<*0.01, ** p*<*0.05, * p*<*0.10.

# Table A10: The Heterogeneous Effects of PLTCI Implementation on the Relationship with Spouse by Race and Ethnicity

(1) (2) (3) (4) (5) (6)

Panel A: Non-Hispanic Black

|  | Criticize | Let You Down | Get on Nerves | Open Up Worries | Rely on Problems | Understand Feelings |
| --- | --- | --- | --- | --- | --- | --- |
| PLTCI implementation | 0.001 | 0.040 | 0.106 | -0.137 | -0.070 | -0.057 |
|  | (0.097) | (0.115) | (0.106) | (0.129) | (0.070) | (0.151) |
| Mean of DV. | 2.146 | 2.089 | 1.766 | 3.430 | 3.726 | 3.158 |
| Number of Obs. | 1,495 | 1,490 | 1,498 | 1,498 | 1,495 | 1,496 |
| Panel B: Non-Hispanic White | | | | | | |
|  | Criticize | Let You Down | Get on Nerves | Open Up Worries | Rely on Problems | Understand Feelings |
| PLTCI implementation | -0.086** | -0.062** | 0.003 | -0.004 | 0.012 | 0.033 |
|  | (0.034) | (0.027) | (0.031) | (0.038) | (0.018) | (0.036) |
| Mean of DV. | 2.134 | 1.956 | 1.760 | 3.441 | 3.780 | 3.322 |
| Number of Obs. | 10,350 | 10,344 | 10,349 | 10,354 | 10,360 | 10,345 |

*Notes*. The table reports the heterogeneous effects of the Partnership Long-Term Care Insurance (PLTCI) implementation on relationships with the spouse of respondents by race, using the working sample of HRS individuals who are age eligible (no more than 65) and health eligible (no Activities of Daily Living (ADL) limitations) during the period in which the program was in place, between 2004 and 2018. Due to the small sample sizes of Hispanic and non-Hispanic individuals of other races in the non-PLTCI states, we excluded these groups from the working sample. Only non-Hispanic White (White) and non-Hispanic Black (Black) individuals are included in the final working sample. The specification in each column includes year and the PLTCI-expansion-group fixed effects. The mean of the dependent variables (DV.) indicates the average of each variable in each column for the states that did not have PLTCI in place. Each column represents a dependent variable measuring a specific aspect of respondents’ relationships with their respective group: *Criticize* (“How much do they criticize you?”), *Let You Down* (“How much do they let you down when you are counting on them?”), *Get on Nerves* (“How much do they get on your nerves?”), *Open Up Worries* (“How much can you open up to them if you need to talk about your worries?”), *Rely on Problems* (“How much can you rely on them if you have a serious problem?”) and *Understand Feelings* (“How much do they really understand the way you feel about things?”). Responses are on a four-point Likert scale from 1 (“Not at all”), 2 (“A little”), 3 (“Some”), and 4 (“A lot”). Standard errors are clustered at the state level.

*** p*<*0.01, ** p*<*0.05, * p*<*0.10.

# Table A11: The Heterogeneous Effects of PLTCI Implementation on the Relationship with Children by Household Wealth

(1) (2) (3) (4) (5) (6)

Panel A: Low (*<* 50 percentile)

|  | Criticize | Let You Down | Get on Nerves | Open Up Worries | Rely on Problems | Understand Feelings |
| --- | --- | --- | --- | --- | --- | --- |
| PLTCI implementation | -0.054 | -0.068 | -0.049 | -0.008 | 0.019 | -0.026 |
|  | (0.058) | (0.065) | (0.072) | (0.073) | (0.068) | (0.044) |
| Mean of DV. | 2.018 | 1.793 | 1.902 | 3.088 | 3.292 | 3.115 |
| Number of Obs. | 5,891 | 5,862 | 5,942 | 5,886 | 5,904 | 5,891 |
| Panel B: High (*>* 50 percentile) | | | | | | |
|  | Criticize | Let You Down | Get on Nerves | Open Up Worries | Rely on Problems | Understand Feelings |
| PLTC implementation | -0.098* | -0.069* | -0.065* | 0.090* | 0.127** | 0.057 |
|  | (0.051) | (0.038) | (0.037) | (0.045) | (0.047) | (0.037) |
| Mean of DV. | 1.978 | 1.742 | 1.807 | 3.003 | 3.297 | 3.095 |
| Number of Obs. | 9,211 | 9,199 | 9,285 | 9,213 | 9,217 | 9,220 |

*Notes*. The table reports the heterogeneous effects of the Partnership Long-Term Care Insurance (PLTCI) implementation on relationships with the children of respondents by household wealth, using the working sample of HRS individuals who are age eligible (no more than 65) and health eligible (no Activities of Daily Living (ADL) limitations) during the period in which the program was in place, between 2004 and 2018. The specification in each column includes year and the PLTCI-expansion-group fixed effects. Low household wealth means that the total wealth is below the 50 percentile (approx. $169,000) of the distribution in the study period in the working sample. The total wealth is defined as the net value of household wealth calculated by subtracting the value of all liabilities from the value of all assets owned by the household. The mean of the dependent variables (DV.) indicates the average of each variable in each column for the states that did not have PLTCI in place. Each column represents a dependent variable measuring a specific aspect of respondents’ relationships with their respective group: *Criticize* (“How much do they criticize you?”), *Let You Down* (“How much do they let you down when you are counting on them?”), *Get on Nerves* (“How much do they get on your nerves?”), *Open Up Worries* (“How much can you open up to them if you need to talk about your worries?”), *Rely on Problems* (“How much can you rely on them if you have a serious problem?”) and *Understand Feelings* (“How much do they really understand the way you feel about things?”). Responses are on a four-point Likert scale from 1 (“Not at all”), 2 (“A little”), 3 (“Some”), and 4 (“A lot”). Standard errors are clustered at the state level.

*** p*<*0.01, ** p*<*0.05, * p*<*0.10.

# Table A12: The Heterogeneous Effects of PLTCI Implementation on the Relationship with Spouse by Household Wealth

|  | (1) | (2) | (3) | (4) | (5) | (6) |
| --- | --- | --- | --- | --- | --- | --- |
| Panel A: Low (*<* 50 percentile) | | | | | | |
|  | Criticize | Let You Down | Get on Nerves | Open Up Worries | Rely on Problems | Understand Feelings |
| PLTCI implementation | 0.040 | 0.030 | 0.062 | -0.094 | -0.051 | -0.048 |
|  | (0.062) | (0.060) | (0.052) | (0.065) | (0.045) | (0.072) |
| Mean of DV. | 2.166 | 1.925 | 1.828 | 3.416 | 3.732 | 3.304 |
| Number of Obs. | 4,505 | 4,496 | 4,510 | 4,515 | 4,517 | 4,508 |
| Panel B: High (*>* 50 percentile) | | | | | | |
|  | Criticize | Let You Down | Get on Nerves | Open Up Worries | Rely on Problems | Understand Feelings |
| PLTCI implementation | -0.110*** | -0.114*** | -0.024 | 0.007 | 0.020 | 0.053 |
|  | (0.040) | (0.042) | (0.036) | (0.036) | (0.021) | (0.039) |
| Mean of DV. | 2.126 | 2.006 | 1.741 | 3.452 | 3.790 | 3.312 |
| Number of Obs. | 8,725 | 8,719 | 8,722 | 8,723 | 8,728 | 8,718 |

*Notes*. The table reports the heterogeneous effects of the Partnership Long-Term Care Insurance (PLTCI) implementation on relationships with the spouse of respondents by household wealth, using the working sample of HRS individuals who are age eligible (no more than 65) and health eligible (no Activities of Daily Living (ADL) limitations) during the period in which the program was in place, between 2004 and 2018. The specification in each column includes year and the PLTCI-expansion-group fixed effects. Low household wealth means that the total wealth is below the 50 percentile (approx. $169,000) of the distribution in the study period in the working sample. The total wealth is defined as the net value of household wealth calculated by subtracting the value of all liabilities from the value of all assets owned by the household. The mean of the dependent variables (DV.) indicates the average of each variable in each column for the states that did not have PLTCI in place. Each column represents a dependent variable measuring a specific aspect of respondents’ relationships with their respective group: *Criticize* (“How much do they criticize you?”), *Let You Down* (“How much do they let you down when you are counting on them?”), *Get on Nerves* (“How much do they get on your nerves?”), *Open Up Worries* (“How much can you open up to them if you need to talk about your worries?”), *Rely on Problems* (“How much can you rely on them if you have a serious problem?”) and *Understand Feelings* (“How much do they really understand the way you feel about things?”). Responses are on a four-point Likert scale from 1 (“Not at all”), 2 (“A little”), 3 (“Some”), and 4 (“A lot”). Standard errors are clustered at the state level.

*** p*<*0.01, ** p*<*0.05, * p*<*0.10.

# Table A13: The Heterogeneous Effects of PLTCI Implementation on the Relationship with Children by LTCI Ownership

|  | (1) | (2) | (3) (4) | | (5) | (6) |
| --- | --- | --- | --- | --- | --- | --- |
|  |  |  | Panel A: With LTCI | |  |  |
|  | Criticize | Let You Down | Get on Nerves Open Up Worries | | Rely on Problems | Understand Feelings |
| PLTCI implementation | -0.052 | 0.052 | -0.236*** 0.280** | | 0.263*** | 0.233*** |
|  | (0.123) | (0.095) | (0.079) (0.117) | | (0.095) | (0.080) |
| Mean of DV. | 1.889 | 1.707 | 1.911 2.854 | | 3.167 | 2.974 |
| Number of Obs. | 1,656 | 1,649 | 1,676 1,657 | | 1,657 | 1,658 |
|  |  |  | Panel B: Without LTCI | |  |  |
|  | Criticize | Let You Down | Get on Nerves | Open Up Worries | Rely on Problems | Understand Feelings |
| PLTCI implementation | -0.074* | -0.070** | -0.039 | 0.043 | 0.074* | 0.009 |
|  | (0.038) | (0.027) | (0.029) | (0.039) | (0.039) | (0.030) |
| Mean of DV. | 2.002 | 1.764 | 1.840 | 3.053 | 3.307 | 3.115 |
| Number of Obs. | 13,245 | 13,211 | 13,349 | 13,242 | 13,262 | 13,251 |

*Notes*. The table reports the heterogeneous effects of the Partnership Long-Term Care Insurance (PLTCI) implementation on relationships with the children of respondents by whether individuals had a Long-Term Care Insurance (LTCI) policy, using the working sample of HRS individuals who are age eligible (no more than 65) and health eligible (no Activities of Daily Living (ADL) limitations) during the period in which the program was in place, between 2004 and 2018. The specification in each column includes year and the PLTCI-expansion-group fixed effects. The mean of the dependent variables (DV.) indicates the average of each variable in each column for the states that did not have PLTCI in place. Each column represents a dependent variable measuring a specific aspect of respondents’ relationships with their respective group: *Criticize* (“How much do they criticize you?”), *Let You Down* (“How much do they let you down when you are counting on them?”), *Get on Nerves* (“How much do they get on your nerves?”), *Open Up Worries* (“How much can you open up to them if you need to talk about your worries?”), *Rely on Problems* (“How much can you rely on them if you have a serious problem?”) and *Understand Feelings* (“How much do they really understand the way you feel about things?”). Responses are on a four-point Likert scale from 1 (“Not at all”), 2 (“A little”), 3 (“Some”), and 4 (“A lot”). Standard errors are clustered at the state level.

*** p*<*0.01, ** p*<*0.05, * p*<*0.10.

# Table A14: The Heterogeneous Effects of PLTCI Implementation on the Relationship with Spouse by LTCI Ownership

|  | (1) | (2) | (3) (4) | | (5) | (6) |
| --- | --- | --- | --- | --- | --- | --- |
|  |  |  | Panel A: With LTCI | |  |  |
|  | Criticize | Let You Down | Get on Nerves Open Up Worries | | Rely on Problems | Understand Feelings |
| PLTCI implementation | -0.182** | -0.217*** | -0.073 -0.013 | | 0.031 | 0.010 |
|  | (0.082) | (0.076) | (0.091) (0.095) | | (0.044) | (0.097) |
| Mean of DV. | 2.132 | 1.947 | 1.780 3.537 | | 3.812 | 3.421 |
| Number of Obs. | 1,554 | 1,555 | 1,558 1,556 | | 1,559 | 1,554 |
|  |  |  | Panel B: Without LTCI | |  |  |
|  | Criticize | Let You Down | Get on Nerves | Open Up Worries | Rely on Problems | Understand Feelings |
| PLTCI implementation | -0.044 | -0.048* | 0.020 | -0.036 | -0.007 | 0.015 |
|  | (0.036) | (0.028) | (0.025) | (0.030) | (0.017) | (0.033) |
| Mean of DV. | 2.140 | 1.978 | 1.771 | 3.430 | 3.766 | 3.296 |
| Number of Obs. | 11,484 | 11,468 | 11,483 | 11,490 | 11,495 | 11,481 |

*Notes*. The table reports the heterogeneous effects of the Partnership Long-Term Care Insurance (PLTCI) implementation on relationships with the spouse of respondents by whether individuals had a Long-Term Care Insurance (LTCI) policy, using the working sample of HRS individuals who are age eligible (no more than 65) and health eligible (no Activities of Daily Living (ADL) limitations) during the period in which the program was in place, between 2004 and 2018. The specification in each column includes year and the PLTCI-expansion-group fixed effects. The mean of the dependent variables (DV.) indicates the average of each variable in each column for the states that did not have PLTCI in place. Each column represents a dependent variable measuring a specific aspect of respondents’ relationships with their respective group: *Criticize* (“How much do they criticize you?”), *Let You Down* (“How much do they let you down when you are counting on them?”), *Get on Nerves* (“How much do they get on your nerves?”), *Open Up Worries* (“How much can you open up to them if you need to talk about your worries?”), *Rely on Problems* (“How much can you rely on them if you have a serious problem?”) and *Understand Feelings* (“How much do they really understand the way you feel about things?”). Responses are on a four-point Likert scale from 1 (“Not at all”), 2 (“A little”), 3 (“Some”), and 4 (“A lot”). Standard errors are clustered at the state level.

*** p*<*0.01, ** p*<*0.05, * p*<*0.10.

# Table A15: Descriptive Statistics of the Leave-Behind Working Sample and General Sample in 2004-2018

Leave-Behind Working Sample HRS General Sample

| N. | | Mean | S.D. |  | N. | Mean | S.D. |  |
| --- | --- | --- | --- | --- | --- | --- | --- | --- |
| Age 15,387 | | 60.45 | 7.78 |  | 137,611 | 66.75 | 11.58 |  |
| **Region of residence (%)** | |  |  |  |  |  |  |  |
| Northeast | 2,517 | 16.36 |  | 21,280 | | 15.46 |  | |
| Midwest | 3,134 | 20.37 |  | 32,118 | | 23.34 |  | |
| South | 5,698 | 37.03 |  | 56,813 | | 41.29 |  | |
| West | 4,038 | 26.24 |  | 27,400 | | 19.91 |  | |
| **Race/Ethnicity (%)** |  |  |  |  | |  |  | |
| White/Caucasian | 11,421 | 74.45 |  | 100,042 | | 72.87 |  | |
| Black/African American/Other | 3,920 | 25.56 |  | 37,250 | | 27.13 |  | |
| **Religion (%)** |  |  |  |  | |  |  | |
| Protestant | 8,603 | 56.03 |  | 82,322 | | 60.00 |  | |
| Catholic | 4,275 | 27.84 |  | 37,167 | | 27.09 |  | |
| Other | 2,475 | 16.13 |  | 17,705 | | 12.91 |  | |
| **Place of Birth (%)** |  |  |  |  | |  |  | |
| US | 13,106 | 85.22 |  | 118,564 | | 86.22 |  | |
| Not US | 2,275 | 14.79 |  | 18,945 | | 13.78 |  | |
| **Female (%)** |  |  |  |  | |  |  | |
| Female | 15,387 | 60.00 |  | 137,611 | | 49.00 |  | |
| **Marital Status (%)** |  |  |  |  | |  |  | |
| Married/Partnered | 11,981 | 77.89 |  | 87,835 | | 63.88 |  | |
| Separated/Divorced | 1,928 | 12.53 |  | 18,007 | | 13.10 |  | |
| Widowed | 1,117 | 7.26 |  | 25,120 | | 18.27 |  | |
| Never Married | 357 | 2.32 |  | 6,555 | | 4.77 |  | |
| Number of Children | 15,387 | 3.10 | 1.76 | 137,611 | | 3.21 | 2.18 | |
| Income | 15,387 | 30,626 | 73,875 | 137,611 | | 16,756 | 46,184 | |
| Years of Education | 15,302 | 13.35 | 2.87 | 137,119 | | 12.51 | 3.29 | |

*Note*. The table presents descriptive statistics for the leave-behind working sample of HRS individuals who were age-eligible (no more than 65) and health eligible (no Activities of Daily Living (ADL) limitations) during the period in which the Partnership Long-Term Care Insurance (PLTCI) program was in place, as well as descriptive statistics for the general HRS sample between 2004 and 2018.

# Table A16: Generalization of The Effect of the PLTCI Implementation on Relationship with Children and Spouse Using Left Behind Weights

|  | (1) | (2) | (3) | (4) | (5) | (6) |
| --- | --- | --- | --- | --- | --- | --- |
| Panel A: Children | | | | | | |
|  | Criticize | Let You Down | Get on Nerves | Open Up Worries | Rely on Problems | Understand Feelings |
| PLTCI implementation | -0.146*** | -0.066** | -0.088** | 0.055 | 0.083 | 0.007 |
|  | (0.038) | (0.030) | (0.041) | (0.054) | (0.050) | (0.035) |
| Mean of DV. | 1.979 | 1.688 | 1.827 | 3.039 | 3.302 | 3.110 |
| Number of Obs. | 13,195 | 13,160 | 13,318 | 13,188 | 13,207 | 13,198 |
|  |  |  |  | Panel B: Spouse |  |  |
|  | Criticize | Let You Down | Get on Nerves | Open Up Worries | Rely on Problems | Understand Feelings |
| PLTCI implementation | -0.138*** | -0.053 | -0.026 | 0.026 | 0.029 | 0.028 |
|  | (0.034) | (0.034) | (0.026) | (0.036) | (0.025) | (0.036) |
| Mean of DV. | 2.148 | 2.097 | 1.691 | 3.433 | 3.766 | 3.316 |
| Number of Obs. | 11,149 | 11,135 | 11,148 | 11,157 | 11,163 | 11,146 |

*Note*. The table reports the effect of the Partnership Long-Term Care Insurance (PLTCI) implementation on relationship with key network of respondents using the working sample of HRS individuals who are age eligible (no more than 65) and health eligible (no Activities of Daily Living (ADL) limitations) during the period in which the program was in place, between 2004 and 2018. The specification in each column includes year and the PLTCI-expansion-group fixed effects and the leave-behind weights are applied in each model. The mean of the dependent variables (DV.) indicates the average of each variable in each column for the states that did not have PLTCI in place. Panel A shows the estimates on relationship with children for individuals that had at least one child in the study period. Panel B shows the estimates on relationship with spouses for individuals that were partnered or married in the study period. Each column represents a dependent variable measuring a specific aspect of respondents’ relationships: *Criticize* (“How much do they criticize you?”), *Let You Down* (“How much do they let you down when you are counting on them?”), *Get on Nerves* (“How much do they get on your nerves?”), *Open Up Worries* (“How much can you open up to them if you need to talk about your worries?”), *Rely on Problems* (“How much can you rely on them if you have a serious problem?”) and *Understand Feelings* (“How much do they really understand the way you feel about things?”). Responses are on a four-point Likert scale from 1 (“Not at all”), 2 (“A little”), 3 (“Some”), and 4 (“A lot”). Standard errors are clustered at the state level. *** p*<*0.01, ** p*<*0.05, * p*<*0.10.

# Table A17: Generalization of The Effect of the PLTCI Implementation on Relationship with Children and Spouse Using Core HRS Weights

|  | (1) | (2) | (3) | (4) | (5) | (6) |
| --- | --- | --- | --- | --- | --- | --- |
| Panel A: Children | | | | | | |
|  | Criticize | Let You Down | Get on Nerves | Open Up Worries | Rely on Problems | Understand Feelings |
| PLTCI implementation | -0.133*** | -0.075** | -0.074* | 0.043 | 0.069 | 0.015 |
|  | (0.038) | (0.029) | (0.036) | (0.049) | (0.044) | (0.032) |
| Mean of DV. | 1.951 | 1.740 | 1.830 | 3.060 | 3.320 | 3.117 |
| Number of Obs. | 13,779 | 13,745 | 13,905 | 13,774 | 13,794 | 13,785 |
|  |  |  |  | Panel B: Spouse |  |  |
|  | Criticize | Let You Down | Get on Nerves | Open Up Worries | Rely on Problems | Understand Feelings |
| PLTCI implementation | -0.133*** | -0.067** | -0.030 | 0.042 | 0.039* | 0.043 |
|  | (0.030) | (0.029) | (0.023) | (0.034) | (0.023) | (0.033) |
| Mean of DV. | 2.147 | 1.980 | 1.762 | 3.433 | 3.767 | 3.332 |
| Number of Obs. | 11,652 | 11,640 | 11,653 | 11,662 | 11,668 | 11,649 |

*Note*. The table reports the effect of the Partnership Long-Term Care Insurance (PLTCI) implementation on relationship with key network of respondents using the working sample of HRS individuals who are age eligible (no more than 65) and health eligible (no Activities of Daily Living (ADL) limitations) during the period in which the program was in place, between 2004 and 2018. The specification in each column includes year and the PLTCI-expansion-group fixed effects and the core HRS weights are applied in each model. The mean of the dependent variables (DV.) indicates the average of each variable in each column for the states that did not have PLTCI in place. Panel A shows the estimates on relationship with children for individuals that had at least one child in the study period. Panel B shows the estimates on relationship with spouses for individuals that were partnered or married in the study period. Each column represents a dependent variable measuring a specific aspect of respondents’ relationships: *Criticize* (“How much do they criticize you?”), *Let You Down* (“How much do they let you down when you are counting on them?”), *Get on Nerves* (“How much do they get on your nerves?”), *Open Up Worries* (“How much can you open up to them if you need to talk about your worries?”), *Rely on Problems* (“How much can you rely on them if you have a serious problem?”) and *Understand Feelings* (“How much do they really understand the way you feel about things?”). Responses are on a four-point Likert scale from 1 (“Not at all”), 2 (“A little”), 3 (“Some”), and 4 (“A lot”). Standard errors are clustered at the state level. *** p*<*0.01, ** p*<*0.05, * p*<*0.10.


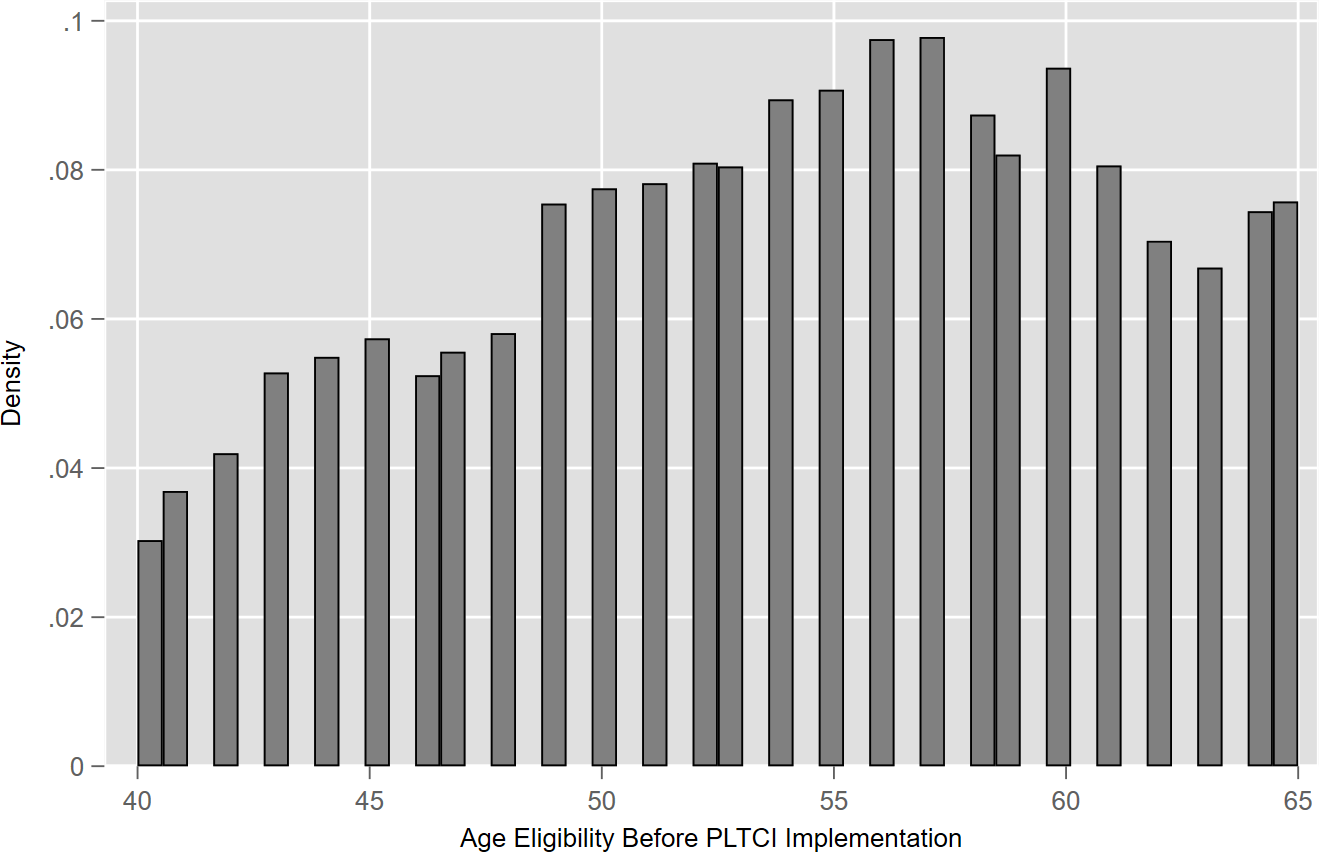


# Figure A1: PLTCI Age Distribution of the Working Sample

*Note*. The graphs display the eligible age distribution for the Partnership Long-Term Care Insurance (PLTCI) among the working sample at the time the PLTCI program was introduced, between 2004 and 2018. PLTCI age eligibility is defined as being no older than 65 years before the implementation of PLTCI.


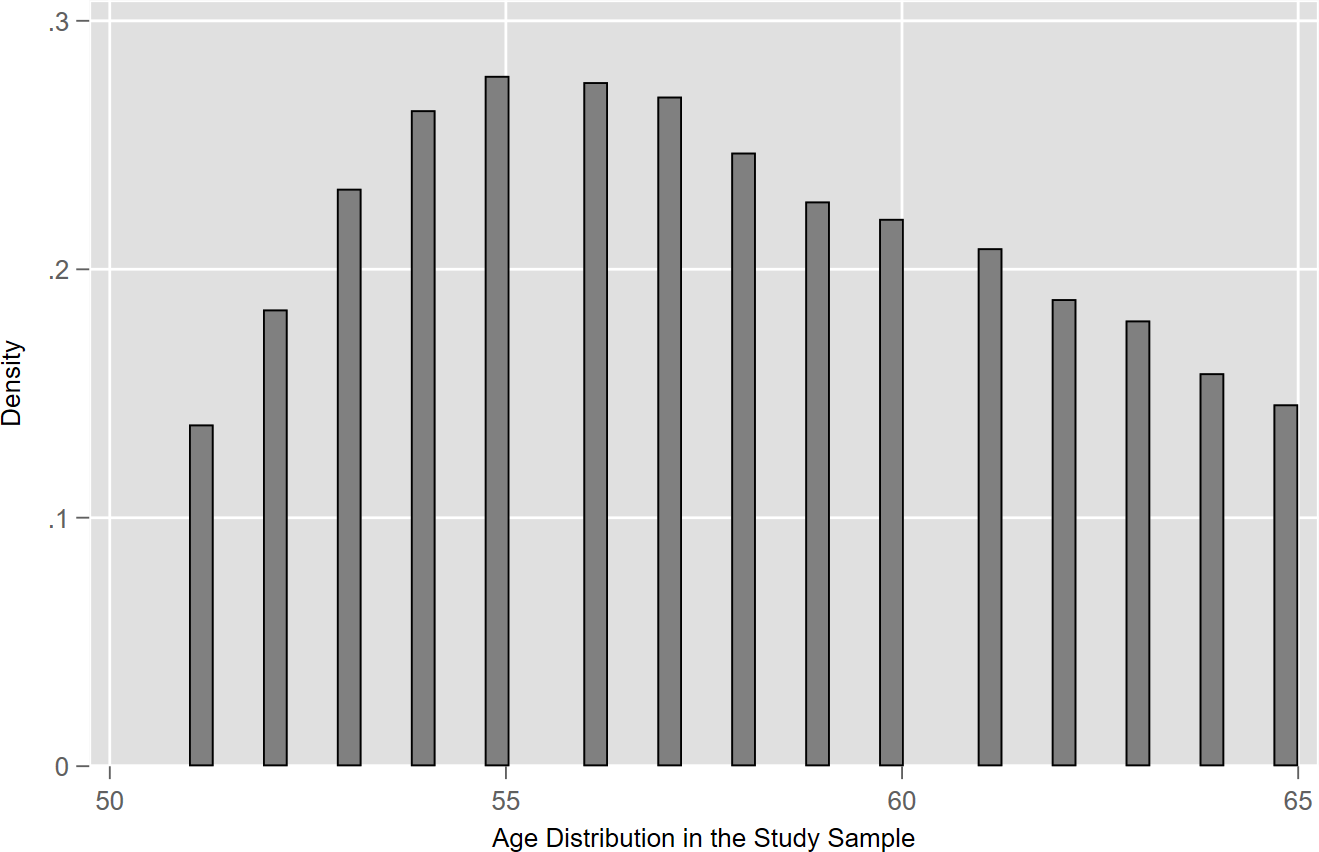


# Figure A2: Age Distribution of the Working Sample

*Note*. The graphs draw the actual age distribution for the working sample of HRS individuals who are age eligible (no more than 65) and health eligible (no Activities of Daily Living (ADL) limitations) during the period in which the Partnership Long-Term Care Insurance (PLTCI) program was in place, between 2004 and 2018.
